# The relationship between childhood asthma and socioeconomic status: a Korean nationwide population-based study

**DOI:** 10.3389/fpubh.2023.1133312

**Published:** 2023-04-25

**Authors:** Won Seok Lee, Jae Kyoon Hwang, Jiin Ryu, Young-Jin Choi, Jae-Won Oh, Chang-Ryul Kim, Man Yong Han, In Hwan Oh, Kyung Suk Lee

**Affiliations:** ^1^Department of Pediatrics, CHA Ilsan Medical Center, CHA University College of Medicine, Goyang, Republic of Korea; ^2^Department of Pediatrics, Hanyang University Guri Hospital, Hanyang University College of Medicine, Guri, Republic of Korea; ^3^Biostatistical Consulting and Research Lab, Medical Research Collaborating Center, Hanyang University, Seoul, Republic of Korea; ^4^Department of Pediatrics, CHA Bundang Medical Center, CHA University College of Medicine, Seongnam, Republic of Korea; ^5^Department of Preventive Medicine, School of Medicine, Kyung Hee University, Seoul, Republic of Korea

**Keywords:** socioeconomic status, asthma, child, public health, prevention

## Abstract

**Purpose:**

This study aimed to investigate associations of socioeconomic status (SES) with asthma exacerbation and asthma-related hospital utilization factors among children with asthma in the Republic of Korea.

**Methods:**

This study retrospectively analyzed population-level data from the Korean National Health Insurance Service, collected from 2013 through 2019. SES was classified into five categories according to the national health insurance premiums quantiles (0 [lowest] to 4 [highest]). The hazard ratios (HRs) for asthma exacerbation, emergency department (ED) visits, hospital admission, and intensive care unit (ICU) admission were analyzed with respect to SES.

**Results:**

Among the five SES groups, SES group 0 (medical aid), had the highest tallies and proportions of children who experienced asthma exacerbations (*n* = 1,682, 4.8%), ED visits (*n* = 932, 2.6%), hospital admission (*n* = 2,734, 7.7%) and ICU admission (*n* = 14, 0.04%). Compared with SES group 4, SES group 0 had adjusted HRs of 3.73 (*p* = 0.0113) and 1.04 (*p* < 0.0001) for ventilator support/tracheal intubation and administration of systemic corticosteroids, respectively. Relative to group 4, the adjusted HRs for ED visits, hospital admission, and ICU admission in group 0 were 1.88 (*p* < 0.0001), 2.20 (*p* < 0.0001), and 7.12 (*p* < 0.0001), respectively. In the survival analysis, group 0 had a significantly higher risk of ED presentation, hospital admission, and ICU admission than the other groups (log-rank *p* < 0.001).

**Conclusion:**

Compared with children of higher SES, those in the lowest SES group had increased risk of asthma exacerbation, hospital admission, and receiving treatment for severe asthma symptoms.

## 1. Introduction

Bronchial asthma is among the commonest chronic inflammatory airway diseases, and its prevalence and incidence continue to increase worldwide (1). It is well known that asthma often begins in childhood and that the incidence is higher among children than adults (2). In the United States (US), the asthma incidence among children younger than 5 years old has been estimated to be 23.4/1000 children per year, contrasted with 4.4/1000/year among youth aged 12–17 years (3).

Overall, there are about 300 million people living with asthma globally, and 100 million new asthma cases will be added by 2025 (4). In the US, about 40 million people have had asthma in their lifetimes (13% of the US population), and 26 million people (8%) are currently living with asthma (5). A Korean study using nationwide cross-sectional data reported estimated asthma prevalences of 0.9% among infants, 2.3% among preschool children, 4.1% among school-aged children, 2.3% among adults, and 4.1% among older adults from 2016 through 2017 (6). The public health significance of asthma lies, in part, in its association with medical resource expenditure, including that required for emergency medical service deployment and hospital admissions, as well as missed school (7). OECD indicated that asthma is a disease of “Avoidable hospital admissions” and asserted that if effective treatment can be delivered, can reduce acute deterioration, and unnecessary admissions (8). Recent study indicate that medical costs due to asthma are significantly higher for people with markers of uncontrolled disease compared with those who do not have asthma (9). In Korea, compared with other age groups, childhood asthma accounts for the largest proportion of asthma-related medical costs (10).

Socioeconomically disadvantaged children are known to experience significantly more acute and chronic illnesses, including asthma, obesity, mental illness and developmental delay, compared with their relatively wealthier counterparts (11). In particular, the prevalence of asthma is higher among children from low-income families, and they tend to have more severe asthma, which has been associated with higher medical costs (12).

We hypothesized that low socioeconomic status (SES) among children might be associated with poorer asthma control, more use of medical services or emergency care. Due to the high prevalence of childhood asthma, a better understanding of the impact of SES on childhood asthma is required to improve quality of life among children with asthma and to reduce medical expenditures.

This study used nationwide population-level data to investigate associations between SES among children with asthma and the likelihood of experiencing clinically diagnosed asthma exacerbations, use of medical services, visits to the emergency department (ED), hospital admissions, intensive care unit (ICU) admissions.

## 2. Methods

### 2.1. Study design and data source

Among 12,961,785 eligible individuals according to the specified age cutoffs (<19 in 2013 and children born between 2013 and 2019), this analysis included children and adolescents (aged ≥2 years and < 19 years at the time of diagnosis) diagnosed with bronchial asthma at any point from 2013 through 2019. Medical records of all subjects were collected from the Korean National Health Insurance Service (NHIS) database. These data included information about demographics, clinical characteristics, treatment, and diagnoses as per the asthma diagnostic details in the *International Classification of Diseases, Tenth Revision* (ICD-10).

This study was approved by the institutional review board of the Hanyang University Guri Hospital (2022–06-038). The requirement for informed consent from participants was waived because all of the NHIS data were anonymized. This retrospective study was performed in accordance with all relevant guidelines and regulations.

### 2.2. Study sample

**Figure 1:**
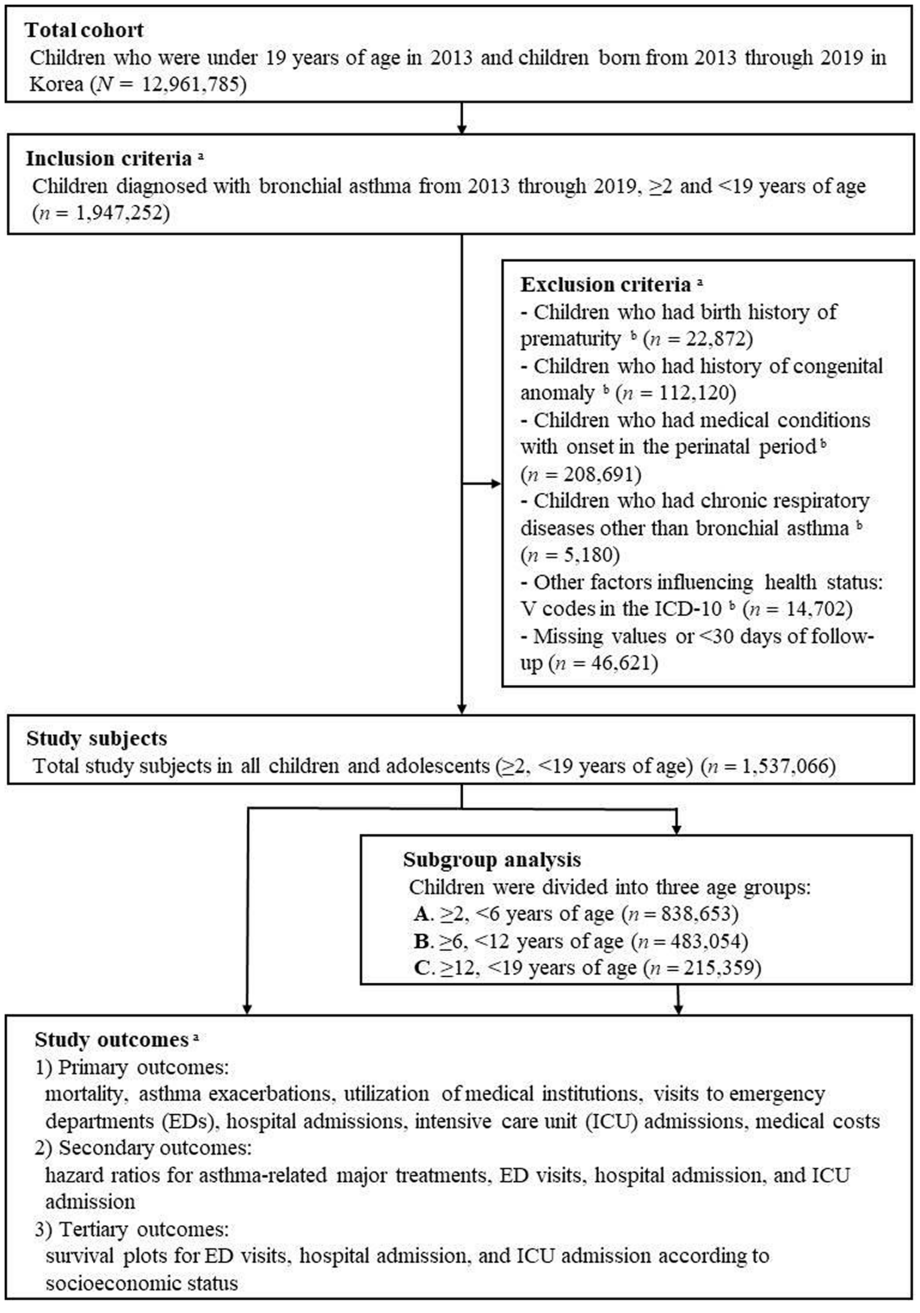
Flow diagram of the cohort, inclusions, exclusions, and outcomes. ^a^Obtained from the Korean National Health Insurance Service (NHIS), ^b^Diagnosis and conditions in children were identified by ICD-10 codes (Supplementary Appendix 1).

A total of 1,947,252 children and adolescents between the ages of 2 and 19 years were diagnosed with bronchial asthma in Korea from 2013 through 2019. The exclusion criteria were a history of preterm birth (*n* = 22,872), a history of congenital anomaly (*n* = 112,120), a diagnosis of a medical condition with onset during the perinatal period (*n* = 208,691), and a chronic respiratory disease diagnosis other than bronchial asthma (*n* = 5,180). We also excluded individuals with ICD-10 V codes (supplementary classification factors influencing health status and contact with health services, *n* = 14,702), as well as those with missing values in their records or with <30 days of follow-up (*n* = 46,621). Finally, 1,537,066 children and adolescents were included in the analysis (Figure 1).

### 2.3. Definitions

Childhood asthma was defined according to the following criteria: (1) age ≥ 2 and < 19 years; (2) at least two claims under ICD-10 codes J45–J46; and (3) at least one claim during the baseline period for prescription of asthma-related drugs, such as inhaled or systemic corticosteroids, bronchodilators, leukotriene receptor antagonists, and xanthine derivatives (13, 14). Children under 2 years of age were excluded because other intrapulmonary airway disorders—such as bronchopulmonary dysplasia and acute viral bronchiolitis—that frequently affect this age group give rise to definitional and diagnostic problems (15).

In terms of SES, study subjects were divided into five groups (rated 0–4). SES 0 comprised children and adolescents on medical aid. Medical aid refers to the public assistance that the state guarantees for the indemnity of medical problems of low-income citizens who do not have the ability to maintain a livelihood or who have difficulties with daily living (16). There are various criteria, but it mainly covers recipients of the National Basic Livelihood Security, and as of 2023, four-person households with monthly incomes less than 1.845 Million Won (almost US$1,581, $1 = 1166.72 Won, Korean Won at the average 2019 exchange rate) are eligible (16, 17, 18).

The rest, excluding the subjects on medical aid, were divided according to the twenty grades of medical insurance costs as followings: SES 1 (grade 1 to 5, lowest), 2 (grade 6 to 10, low to middle), 3 (grade 11 to 15, middle to high), and 4 (grade 16 to 20 of medical insurance costs, highest).

### 2.4. Primary, secondary, and tertiary outcomes

The primary outcomes were mortality, asthma exacerbation, use of medical services, visits to EDs, hospital admissions, and ICU admissions of childhood asthma according to SES. The secondary outcomes were hazard ratios (HRs) for asthma-related major treatment, ED visits, hospital admission, and ICU admission. Major treatments comprised ventilator support or tracheal intubation, as well as administration of systemic corticosteroids. The tertiary outcomes were survival plots for ED visits, hospital admission, and ICU admission.

### 2.5. Statistical analysis

Baseline characteristics (age, sex, insurance type, and residence), allergy history (except, bronchial asthma), and asthma medications were compared among the SES groups using the McNemar test. HRs with 95% confidence intervals (CIs) were calculated for the outcomes using a Cox proportional hazards regression. The Kaplan–Meier method was used to estimate survival curves during follow-up, and survival was compared among the groups using the log-rank test. All analyses were conducted using SAS, version 9.4 (SAS Institute, Cary, NC, United States). All tests were two-sided, and *value of p*s <0.05 were considered statistically significant.

## 3. Results

### 3.1. Baseline characteristics

More than half of the eligible study subjects were between 2 and 6 years of age (*n* = 838,653, 54.6%), compared with 215,359 (14.0%) who were between 12 and 19 years old (Table 1). There were 35,292 children and adolescents (2.3%) in SES group 0. Table 1 also summarizes asthma medications during follow-up.

**Table 1:** Demographic and clinical characteristics of the participants in the main cohort.^a^

	Total no. (%), (*N* = 1,537,066)^a^	Social economic status (SES, 0 to 4)					*p* value
		0 (Medical aid) no. (%), (*N* = 35,292)^a^	1 (Lowest) no. (%), (*N* = 181,756)^a^	2 (Low to Middle) no. (%), (*N* = 241,103)^a^	3 (Middle to High) no. (%), (*N* = 498,685)^a^	4 (Highest) no. (%), (*N* = 580,230)^a^	
1. Baseline characteristics^b^							
1) Age, mean (yr)	6.4 ± 4.3	9.0 ± 5.1	7.0 ± 4.6	6.1 ± 4.3	5.6 ± 3.8	6.7 ± 4.3	
2) Age group (*n*)							<0.0001
≥2, <6 year	838,653 (54.6)	11,768 (33.3)	87,328 (48.0)	139,491 (57.9)	310,670 (62.3)	289,396 (49.9)	
≥6,<12 year	483,054 (31.4)	11,534 (32.7)	61,069 (33.6)	69,986 (29.0)	140,882 (28.3)	199,583 (34.4)	
≥12, <19 year	215,359 (14.0)	11,990 (34.0)	33,359 (18.4)	31,626 (13.1)	47,133 (9.5)	91,251 (15.7)	
3) Sex							<0.0001
Male	811,729 (52.8)	17,935 (50.8)	96,005 (52.8)	126,603 (52.5)	260,560 (52.2)	310,626 (53.5)	
Female	725,337 (47.2)	17,357 (49.2)	85,751 (47.2)	114,500 (47.5)	238,125 (47.8)	269,604 (46.5)	
4) Type of insurance							<0.0001
Self-employed health insurance	350,244 (22.8)	–	38,368 (21.1)	78,571 (32.6)	122,164 (24.5)	111,141 (19.2)	
Employed health insurance	1,151,530 (74.9)	–	143,388 (78.9)	162,532 (67.4)	376,521 (75.5)	469,089 (80.8)	
Medical aid	35,292 (2.3)	35,292 (100.0)	–	–	–	–	
5) Residence							<0.0001
Seoul	263,949 (17.2)	4,516 (12.8)	26,917 (14.8)	37,395 (15.5)	74,534 (14.9)	120,587 (20.8)	
Metropolitan	398,250 (25.9)	11,363 (32.2)	48,867 (26.9)	63,561 (26.4)	133,054 (26.7)	141,405 (24.4)	
Urban	791,801 (51.5)	16,175 (45.8)	92,235 (50.7)	123,599 (51.3)	262,586 (52.7)	297,206 (51.2)	
Rural	83,066 (5.4)	3,238 (9.2)	13,737 (7.6)	16,548 (6.9)	28,511 (5.7)	21,032 (3.6)	
2. Allergy history, except bronchial asthma^b^							
1) Allergic rhinitis	1,160,865 (75.5)	29,129 (82.5)	140,836 (77.5)	179,938 (74.6)	362,233 (72.6)	448,729 (77.3)	<0.0001
2) Atopic dermatitis	525,733 (34.2)	11,389 (32.3)	61,659 (33.9)	82,949 (34.4)	174,547 (35.0)	195,189 (33.6)	<0.0001
3. Asthma medications during follow-up^b^							
1) Any Intranasal corticosteroids (ICS)	736,400 (47.9)	15,399 (43.6)	83,767 (46.1)	116,504 (48.3)	250,386 (50.2)	270,344 (46.6)	<0.0001
2) Systemic corticosteroids	662,826 (43.1)	15,266 (43.3)	78,022 (42.9)	105,946 (43.9)	219,088 (43.9)	244,504 (42.1)	<0.0001
3) Leukotriene receptor antagonists (LTRA)	877,807 (57.1)	18,708 (53.0)	100,840 (55.5)	135,384 (56.2)	287,951 (57.7)	334,924 (57.7)	<0.0001
4) Long-acting beta-2 agonists (LABA)	1,227,848 (79.9)	25,169 (71.3)	141,623 (77.9)	196,028 (81.3)	414,430 (83.1)	450,598 (77.7)	<0.0001
5) Shot-acting beta-2 agonists (SABA)	811,096 (52.8)	17,613 (49.9)	93,899 (51.7)	129,370 (53.7)	273,247 (54.8)	296,967 (51.2)	<0.0001
6) Anticholinergics	22,675 (1.5)	906 (2.6)	3,078 (1.7)	3,718 (1.5)	6,679 (1.3)	8,294 (1.4)	<0.0001
7) Xanthines	370,150 (24.1)	10,158 (28.8)	45,418 (25.0)	60,526 (25.1)	120,671 (24.2)	133,377 (23.0)	<0.0001
8) Only LABA	1,215,410 (79.1)	24,466 (69.3)	139,684 (76.9)	194,204 (80.5)	411,752 (82.6)	445,304 (76.7)	<0.0001

a Values are reported as *n* (%) unless otherwise indicated.

b Obtained from Korean National Health Insurance Service (NHIS) data.

### 3.2. Primary outcomes: Asthma exacerbation, utilization of or admission to medical institutions

During the period under study, none of the subjects died from asthma. Across the entire age eligibility range, the proportion of study subjects who experieced asthma exacerbations was highest in SES group 0 (*n* = 1,682, 4.8%). Group 0 also had the highest tallies and proportions of annual ED visits (*n* = 932, 2.6%), hospital admissions (*n* = 2,734, 7.7%), and ICU admissions (*n* = 14, 0.04%), with statistically significant differences from the other groups (Table 2).

**Table 2:** Asthma exacerbation, utilization of or admissions to medical institutions, and medical costs due to childhood asthma (≥2 and < 19 years of age).

	Total No. (%), (*N* = 1,537,066)^a^	Socioeconomic status (SES, 0 to 4)					*p*-value
		0 (Medical aid) No. (%), (*n* = 35,292)^a^	1 (Lowest) No. (%), (*n* = 181,756)^a^	2 (Low to Middle) No. (%), (*n* = 241,103)^a^	3 (Middle to High) No. (%), (*n* = 498,685)^a^	4 (Highest) No. (%), (*n* = 580,230)^a^	
1. Asthma exacerbation^b^							
Dianosis of asthma exacerbations	52,754 (3.4%)	1,682 (4.8%)	6,809 (3.7%)	9,064 (3.8%)	17,316 (3.5%)	17,883 (3.1%)	<0.0001
Events numbers of asthma exacerbations	182,154	6,336	22,893	30,902	60,244	61,779	
Annual number of asthma exacerbations (*n*/100,000 persons)	2,481	3,679	2,627	2,660	2,548	2,229	
2. Asthma-related hospital utilization^b^							
Primary hospital visits	942,289 (61.3%)	21,256 (60.2%)	111,940 (61.6%)	146,402 (60.7%)	301,649 (60.5%)	361,042 (62.2%)	
Secondary hospital visits	832,669 (54.2%)	18,880 (53.5%)	97,873 (53.8%)	130,205 (54.0%)	270,630 (54.3%)	315,081 (54.3%)	
Tertiary hospital visits	742,524 (48.3%)	16,883 (47.8%)	86,703 (47.7%)	116,741 (48.4%)	244,245 (49.0%)	277,952 (47.9%)	
ED visits, patient (*n*)	24,825 (1.6%)	932 (2.6%)	3,203 (1.8%)	4,238 (1.8%)	7,817 (1.6%)	8,635 (1.5%)	<0.0001
Admissions, patient (*n*)	84,164 (5.5%)	2,734 (7.7%)	10,737 (5.9%)	15,462 (6.4%)	29,597 (5.9%)	25,634 (4.4%)	<0.0001
ICU admission, patient (*n*)	104 (0.01%)	14 (0.04%)	19 (0.01%)	17 (0.01%)	22 (0.00%)	32 (0.01%)	<0.0001
Annual primary hospital visits, (*n*/100,000 persons)	31,787	29,127	31,855	30,711	31,059	33,061	<0.0001
Annual Secondary hospital visits, (*n*/100,000 persons)	22,737	21,284	22,317	22,326	23,037	22,882	<0.05
Annual tertiary hospital visits, (*n*/100,000 persons)	17,762	16,794	17,254	17,602	18,369	17,543	<0.0001
Annual ED visits, patient, (*n*/100,000 persons)	332	529	360	357	324	306	<0.0001
Annual hospitalizations, (*n*/100,000 persons)	1,159	1,624	1,247	1,353	1,269	929	<0.0001
Annual ICU admission, patient, (*n*/100,000 persons)	1	8	2	1	1	1	<0.0001

ED, emergency department; ICU, intensive care units.

a Values are reported as *n* (%) unless otherwise indicated.

b Obtained from Korean National Health Insurance Service (NHIS) data.

SES group 0 had fewer annual primary, secondary, and tertiary hospital visits than the other SES groups (*n* = 29,127, *n* = 21,284, and *n* = 16,794/100,000 persons, respectively). However, group 0 had significantly more annual ED visits, hospitalizations, and ICU admissions (Table 2).

Supplementary Table 1 summarizes the three age-group–stratified (2–5, 6–11, and 12–18 years of age) comparisons of outcomes between the different SES groups. The outcomes of interest were asthma exacerbations and usage of or admissions to medical institutions due to asthma.

### 3.3. Secondary outcomes: HRs for major treatment, ED visits, hospital admission, and ICU admission

Relative to SES group 4, the adjusted HRs for ventilator support or tracheal intubation, and administration of systemic corticosteroids in SES group 0 were 3.73 (95% CI 1.35–10.35, *p* = 0.0113) and 1.04 (95% CI 1.04–1.06, *p* < 0.0001), respectively. The adjusted HRs for ED visits, hospital admission, and ICU admission in group 0 were 1.88 (95% CI 1.75–2.01, *p* < 0.0001), 2.20 (95% CI 2.11–2.28, *p* < 0.0001), and 7.12 (95% CI 3.72–13.62, *p* < 0.0001), compared with group 4, respectively. The adjusted HRs for ventilator support or tracheal intubation, administration of systemic corticosteroids, ED visits, hospital admission, and ICU admission in group 1 were also statistically significant relative to group 4 (Table 3).

**Table 3:** Crude and adjusted hazard ratio (HR) of major treatments, emergency department visits, and admission to hospital and ICU in children with bronchial asthma.

	Ventilator support or tracheal intubation^a^					Systemic corticosteroid^a^					Emergency department (ED) visits^a^				
	No. (%)	cHR (95% CI)	*p*-value	aHR^b^ (95% CI)	*p*-value	No. (%)	cHR (95% CI)	*p*-value	aHR^b^ (95% CI)	*p*-value	No. (%)	cHR (95% CI)	*p*-value	aHR^b^ (95% CI)	*p*-value
SES 0 (*n* = 35,292)	5 (0.01)	4.71 (1.74–12.77)	0.0023	3.73 (1.35–10.35)	0.0113	15,266 (43.3)	1.02 (1.00–1.03)	0.0439	1.04 (1.02–1.06)	<0.0001	932 (2.6)	1.75 (1.63–1.87)	<0.0001	1.88 (1.75–2.01)	<0.0001
SES 1 (*n* = 181,756)	12 (0.01)	2.24 (1.07–4.69)	0.0324	2.15 (1.03–4.51)	0.0420	78,022 (42.9)	1.02 (1.01–1.03)	<0.0001	1.02 (1.01–1.03)	<0.0001	3,203 (1.8)	1.18 (1.13–1.23)	<0.0001	1.19 (1.14–1.24)	<0.0001
SES 2 (*n* = 241,103)	5 (0.00)	0.70 (0.26–1.90)	0.4855	0.74 (0.27–2.02)	0.5569	105,946 (43.9)	1.05 (1.04–1.06)	<0.0001	1.04 (1.03–1.05)	<0.0001	4,238 (1.8)	1.17 (1.13–1.22)	<0.0001	1.15 (1.11–1.19)	<0.0001
SES 3 (*n* = 498,685)	14 (0.00)	0.96 (0.47–1.95)	0.9135	1.10 (0.54–2.26)	0.7850	219,088 (43.9)	1.05 (1.05–1.06)	<0.0001	1.04 (1.04–1.05)	<0.0001	7,817 (1.6)	1.06 (1.03–1.09)	0.0004	1.04 (1.01–1.07)	0.0099
SES 4 (*n* = 580,230)	17 (0.00)	Ref		Ref		244,504 (42.1)	Ref		Ref		8,635 (1.5)	Ref		Ref	
	**Admission to hospital** ^**a**^					**Admission to ICU** ^**a**^									
SES 0 (*n* = 35,292)	2,734 (7.7)	1.76 (1.69–1.83)	<0.0001	2.20 (2.11–2.28)	<0.0001	14 (0.0)	7.02 (3.75–13.16)	<0.0001	7.12 (3.72–13.62)	<0.0001					
SES 1 (*n* = 181,756)	10,737 (5.9)	1.34 (1.31–1.38)	<0.0001	1.37 (1.34–1.40)	<0.0001	19 (0.0)	1.89 (1.07–3.33)	0.0281	1.85 (1.05–3.27)	0.0333					
SES 2 (*n* = 241,103)	15,462 (6.4)	1.46 (1.43–1.49)	<0.0001	1.34 (1.31–1.36)	<0.0001	17 (0.0)	1.27 (0.71–2.29)	0.4274	1.23 (0.68–2.22)	0.4985					
SES 3 (*n* = 498,685)	29,597 (5.9)	1.36 (1.34–1.38)	<0.0001	1.21 (1.19–1.24)	<0.0001	22 (0.0)	0.80 (0.47–1.38)	0.4272	0.83 (0.48–1.43)	0.4899					
SES 4 (*n* = 580,230)	25,634 (4.4)	Ref		Ref		32 (0.0)	Ref		Ref						

cHR, crude hazard ratio; aHR, adjusted hazard ratio; ED, emergency department; ICU, intensive care unit; Ref, reference.

a Obtained from Korean National Health Insurance Service (NHIS) data.

b adjusted by age, sex, type of insurance, allergy history, respiratory disease (bronchiolitis, croup, pneumonia, and Mycoplasma pneumoniae infections).

The crude and adjusted HRs for ventilator support or tracheal intubation, administration of systemic corticosteroids, ED visits, hospital admission, and ICU admission in SES groups 0, 1, and 2 groups relative to the SES 4 group, according to age group (2–5, 6–11, and 12–18 years) are shown in Supplementary Table 2.

### 3.4. Survival plots for ED visits, hospital admission, and admission to ICU

**Figure 2:**
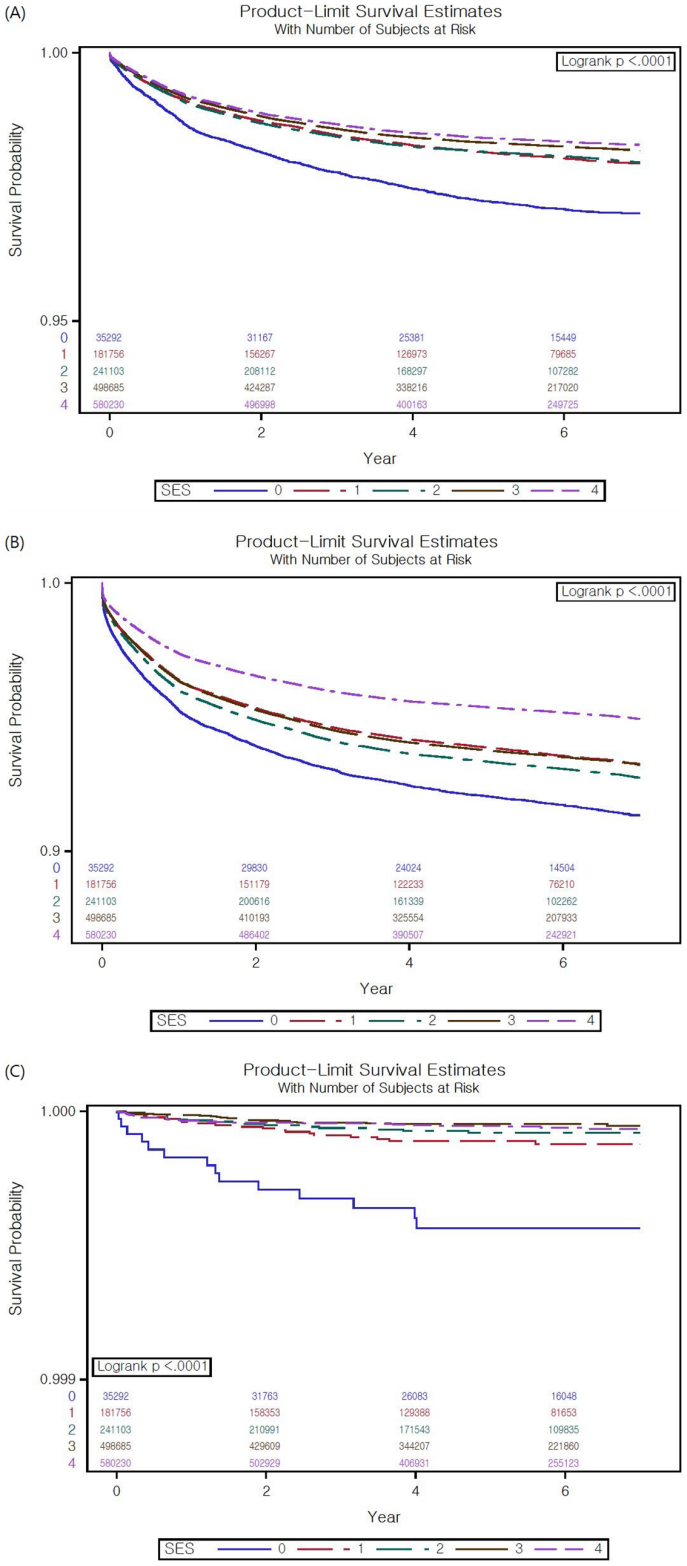
Survival plots for emergency department (ED) visits, hospital admission, and intensive care unit (ICU) admission. **(A)** Survival plot for ED visits. For the visits to ED, the medical aid group (SES 0) had a significantly lower survival probability than the other groups (*p* < 0.001). **(B)** Survival plot for hospital admission. For hospital admission, the medical aid group had the lowest survival probability (*p* < 0.001). **(C)** Survival plot for ICU admission. The medical aid group had a significantly lower survival probability than the other groups in terms of ICU admission (*p* < 0.001).

The survival plots for ED visits, hospital admission, and ICU admission are shown in Figure 2. Group 0 had a significantly lower survival probability than the other SES groups during follow-up (log-rank *p* < 0.001). In terms of hospital admission, group 4 had a significantly higher survival probability than the other groups (*p* < 0.001), and group 0 had the lowest survival probability (*p* < 0.001). The ICU admission survival curves indicate that group 0 had a significantly lower survival probability than the other SES groups (*p* < 0.001).

## 4. Discussion

This study demonstrated that asthma exacerbations, medical facility use, and admissions associated with asthma were significantly more common among children of lower SES. Specifically, interventions for severe asthma, such as ventilator support or tracheal intubation, administration of systemic corticosteroids, ED visits, hospital admissions, and ICU admissions were applied significantly more frequently for children of lower SES, particularly those in the medical aid group. These findings emphasize the importance of more careful consideration SES when treating children with asthma, with more effective management of social spending and utilization of public support.

Several previous studies have reported that children from low-income families tend to have more severe asthma (19, 20). As asthma is often not well controlled among children of lower SES (12), symptoms may be severe, increasing the risk of asthma exacerbations and hospital admission can. Our study demonstrated that children of lower SES had significantly more asthma exacerbations, treatments for severe asthma, as well as more frequent ED presentations, hospitalizations, and ICU admissions. In particular, adjusted HRs of ICU admission in the medical aid group were conspicuously increased. In a previous study conducted in the United States, participants with lower income had higher rates of both asthma treatment failure (rate ratio 1.6, *p* = 0.03) and exacerbation (rate ratio 1.9, *p* = 0.02) (21). A Welsh study using a national cohort also demonstrated that the most socioeconomically deprived patients had more asthma-related accident-and-emergency attendances (incidence rate ratio [IRR] 1.27, *p* = 0.001), more asthma-related emergency admissions (IRR = 1.56, *p* < 0.001), longer asthma-related hospital stays (IRR = 1.64, *p* < 0.001), and were at higher risk of asthma-related death (risk ratio of deaths with any mention of asthma 1.56, *p* = 0.002) (22).

Previous research has linked poor asthma control with avoidable morbidity or mortality, ED visits, hospital admissions (23, 24). Additionally, well controlled asthma has been associated with reduced visits to acute care centers and better quality of life (25, 26).

Meanwhile, aside from asthma severity and emergency treatments, few have investigated the association between SES and the development of asthma. A cohort study in Western Australia observed that children in low-income families since birth had a 2 times higher risk than other children of developing bronchial asthma by age 14 years (27). Chen et al. showed that the likelihood of developing asthma was lower among children whose families had moved up in income than among children who continued to live in low-income families (28). On the other hand, children in low-income families experience higher rates of exposure to endotoxins and infections, which may protect against asthma development (29). More research is needed to clarify the association between SES and the development of bronchial asthma in children.

We found that children in the medical aid group (SES group 0) were significantly less frequently prescribed inhaled corticosteroids than the other group. Previous research has also demonstrated an association between lower SES and underutilization of asthma controllers among children (30). A Canadian study showed that children were less likely to receive inhaled corticosteroids if they came from low-income families. After adjusting for insurance type and disease severity, asthma controller use was the main mechanism through which SES affects asthma control (31). Adherence can be associated with the beliefs that parents have about medications, perceived requirements for treatment, understanding about medications or environmental factors, all of which may be related to SES (32). This may contribute to underutilization of asthma controllers in low-income segments of the population.

Intranasal corticosteroids, leukotriene receptor antagonists, long-acting and short-acting beta-2 agonists are mainly used for asthma control and relief according to available guidelines for asthma management (1). The prescription rates of these medications were relatively low in SES group 0 than higher SES group in this study. This is another indicator that, childhood asthma can be better managed and well controlled with access to higher income levels (33), and low SES predicted poorer medication adherence in asthmatic children (34). In addition, it is well known that the asthma prevalence decreases with age among children and adolescents (3). However, among our study subjects, in SES group 0, asthma diagnoses did not decrease between the ages of 12 and 19 years compared other age groups in medical aid. On the other hand, asthma diagnoses significantly decreased with increasing age in the higher SES group. Basically, the prevalence of childhood asthma peak at school age, and it appears to decline during adolescence (35). SES seems to have an additional influence on this natural pattern of asthma prevalence. Several studies have shown the prevalence of asthma to be higher in lower SES groups (20). One explanation for this is that low-income patients have more doctor contacts than high-income patients (36). Moreover, economically poor living areas are often highly concentrated in air pollutants, such as particulate matter, nitric oxides, and ozone, which are associated with asthma morbidity (37). Additionally, second-hand smoke, indoor pollutant levels, and social stresses are also more prevalent or prominent in lower socioeconomic contexts, contributing to the higher asthma prevalence in lower socioeconomic contexts compared with more affluent settings (37).

The impact of SES on child health may go beyond pure income effects to include factors related to social structure, familial characteristics, and the need and use of health resources (11). SES can directly affect access to medical services (38). Familial factors can also have various relevant effects; for example, whether both parents are employed, whether the family is a single-parent household, and whether the parents have illnesses or disabilities that may affect childrearing (11, 32). Meanwhile higher family affluence has been strongly associated with higher levels of adolescent physical activity (32). Adolescents with lower cardiorespiratory fitness have been shown to have poorer lung function and a higher risk of asthma (39). Bronchial asthma is a chronic disease that requires life-long control measures and regular monitoring; it can be expected that the control and management of asthma is more likely to be poorly supported among children from lower-income families (11, 32).

South Korea is known to have a well-developed and robust public health insurance system. However, our study paradoxically revealed that, despite the children in SES group 0 having more severe asthma and poorer control. The healthcare inequality associated with childhood asthma cannot be resolved simply by subsidizing medical expenses. Improvements in public policy are needed so that social spending on childhood asthma can be more effectively distributed to among children from families with lower income levels. Moreover, continuous management and control of childhood asthma; improvements in living conditions; and education for individual children, parents, and schools should be carried out at the public level.

To the best of our knowledge, this was the first study to use nationwide population-level health data to evaluate the associations between the usage of medical services for childhood asthma according to SES in Korea. Most previous studies about the relationship between bronchial asthma and SES have focused on adult patients, and some of these studies investigated relatively subjective variables representing SES (7, 9, 10). This study classified SES according to the insurance co-payments, which directly reflects familial income levels in South Korea. Additionally, this study used a novel approach to analyze the association between SES and the more comprehensive aspects of childhood asthma—such as emergency treatments (ventilator support or tracheal intubation), asthma exacerbations, use of medical services, visits to the ED, hospital admissions, and ICU admissions—by using a validated national medical insurance database.

This study has several limitations. First, asthma diagnoses were identified according to ICD-10 disease codes in a national health database. Due to limitations of the health data system, more detailed information about asthma severity and control were not available. Second, to minimize bias, we tried to exclude subjects with histories of prematurity, congenital anomalies, and chronic respiratory diseases other than asthma. However, we could not control or adjust for additional factors that can affect asthma severity, such as various underlying diseases, intermittent infectious diseases, and disease control. Third, there was no information about parental factors in the NHIS; therefore, we could not investigate the aforementioned parental or familial factors that may affect asthma control.

In conclusion, asthma exacerbations, medical service use, ED visits, hospital admissions, and ICU admissions were more frequent in association with low SES among children with asthma, despite this group receiving a relatively smaller share of social medical expenditure. Policy improvements are needed so that social spending can be more effectively distributed to children from lower-income families to improve living conditions, as well as optimize educational interventions for children, parents, and schools.

## Prior presentations

This manuscript has not been published earlier in any journal and is not being considered for publication elsewhere.

## Institutional review board statement

The study was conducted according to the guidelines of the Declaration of Helsinki and was approved by the institutional review board of Hanyang University Guri hospital (IRB number 2022-06-038).

## Informed consent statement

The requirement for informed consent from participants was waived, as all data from the Korean National Health Insurance Service (NHIS) were anonymized.

## Data availability statement

The original contributions presented in the study are included in the article/Supplementary material, further inquiries can be directed to the corresponding author.

## Author contributions

KL contributed as corresponding author to this work. WL and JH were 1st co-authors and contributed equally to this work. KL and WL conceived the idea. JR analyzed the data. WL and JH wrote the manuscript with input from all the authors. Y-JC, J-WO, C-RK, MH, and IO investigated and supervised the findings of this study. All authors contributed to the article and approved the submitted version.

## Funding

This work was supported by the Hanyang University Medical Center Development Fund.

## Conflict of interest

The authors declare that the research was conducted in the absence of any commercial or financial relationships that could be construed as a potential conflict of interest.

## Publisher’s note

All claims expressed in this article are solely those of the authors and do not necessarily represent those of their affiliated organizations, or those of the publisher, the editors and the reviewers. Any product that may be evaluated in this article, or claim that may be made by its manufacturer, is not guaranteed or endorsed by the publisher.
