# Supplementary material for: The relationship between childhood asthma and socioeconomic status: a Korean nationwide population-based study
Source: Front Public Health. 2023 Apr 25;11:1133312. doi: 10.3389/fpubh.2023.1133312 (PMC10167280; doi:10.3389/fpubh.2023.1133312)
Supplement: Supplementary file 1 [file Table_1.DOCX]

**Supplementary Tables**

**Supplementary Table 1**. Asthma exacerbation, utilization or admissions of medical institutions, and medical costs due to childhood asthma, divided by age groups

**A.** ≥2, <6 years of age

|  | **Total**  No. (%), (*n* = 838,653) |  | **Socioeconomic status (SES, 0 to 4)** | | | | |  | ***p*-value** |
| --- | --- | --- | --- | --- | --- | --- | --- | --- | --- |
|  |  |  | **0 (Medical aid)** No. (%),  (*n* = 11,768) | **1 (Lowest)**  No. (%),  (*n* = 87,328) | **2 (Low to Middle)** No. (%),  (*n* = 139,491) | **3 (Middle to High)** No. (%),  (*n* = 310,670) | **4 (Highest)**  No. (%),  (*n* = 289,396) |  |  |
| **1. Asthma exacerbation** |  |  |  |  |  |  |  |  |  |
| Dianosis of asthma exacerbations | 28,360 (3.4%) |  | 493 (4.2%) | 3,211 (3.7%) | 5,250 (3.8%) | 10,733 (3.5%) | 8,673 (3.0%) |  | <.0001 |
| Events numbers of asthma exacerbations | 97,944 |  | 1,472 | 10,945 | 17,830 | 37,766 | 29,931 |  |  |
| Annual number of asthma exacerbations  (n/100,000 persons) | 2,571 |  | 2,602 | 2,743 | 2,765 | 2,694 | 2,291 |  |  |
| **2. Asthma-related hospital utilization** |  |  |  |  |  |  |  |  |  |
| Primary hospital visits | 488,026 (58.2%) |  | 6,820 (58.0%) | 50,922 (58.3%) | 80,064 (57.4%) | 179,676 (57.8%) | 170,544 (58.9%) |  |  |
| Secondary hospital visits | 455,908 (54.4%) |  | 6,419 (54.5%) | 47,331 (54.2%) | 75,164 (53.9%) | 168,567 (54.3%) | 158,427 (54.7%) |  |  |
| Tertiary hospital visits | 423,054 (50.4%) |  | 6,083 (51.7%) | 43,885 (50.3%) | 70,064 (50.2%) | 157,035 (50.5%) | 145,987 (50.4%) |  |  |
| ED visits, patient (n) | 13,870 (1.7%) |  | 308 (2.6%) | 1,595 (1.8%) | 2,524 (1.8%) | 4,941 (1.6%) | 4,502 (1.6%) |  | <.0001 |
| Admissions, patient (n) | 59,245 (7.1%) |  | 1,214 (10.3%) | 6,833 (7.8%) | 11,353 (8.1%) | 22,577 (7.3%) | 17,268 (6.0%) |  | <.0001 |
| ICU admission, patient (n) | 47 (0.0%) |  | 3 (0.0%) | 7 (0.0%) | 11 (0.0%) | 11 (0.0%) | 15 (0.0%) |  | 0.0115 |
| Annual primary hospital visits, (n/100,000 persons) | 29,648 |  | 27,307 | 29,562 | 28,142 | 29,427 | 30,798 |  | <.05 |
| Annual Secondary hospital visits, (n/100,000 persons) | 24,317 |  | 22,887 | 24,088 | 23,367 | 24,363 | 24,879 |  | <.05 |
| Annual tertiary hospital visits, (n/100,000 persons) | 20,439 |  | 19,951 | 20,220 | 19,809 | 20,622 | 20,644 |  |  |
| Annual ED visits, patient, (n/100,000 persons) | 358 |  | 537 | 392 | 384 | 346 | 339 |  | <.0001 |
| Annual hospitalizations, (n/100,000 persons) | 1,595 |  | 2,272 | 1,766 | 1,818 | 1,652 | 1,346 |  | <.0001 |
| Annual ICU admission, patient, (n/100,000 persons) | 1 |  | 5 | 2 | 2 | 1 | 1 |  |  |

ED, emergency department; ICU, intensive care unit; ^a^ Values are reported as *n* (%) unless otherwise indicated; ^b^ Obtained from Korean National Health Insurance Service (NHIS) data

**B.** ≥6,<12 years of age

|  | **Total**  No. (%), (*n* = 483,054) |  | **Socioeconomic status (SES, 0 to 4)** | | | | |  | ***p*-value** |
| --- | --- | --- | --- | --- | --- | --- | --- | --- | --- |
|  |  |  | **0 (Medical aid)** No. (%),  (*n* = 11,534) | **1 (Lowest)**  No. (%),  (*n* = 61,069) | **2 (Low to Middle)** No. (%),  (*n* = 69,986) | **3 (Middle to High)** No. (%),  (*n* = 140,882) | **4 (Highest)**  No. (%),  (*n* = 199,583) |  |  |
| **1. Asthma exacerbation** |  |  |  |  |  |  |  |  |  |
| Dianosis of asthma exacerbations | 15,945 (3.3%) |  | 488 (4.2%) | 2,188 (3.6%) | 2,470 (3.5%) | 4,734 (3.4%) | 6,065 (3.0%) |  | <.0001 |
| Events numbers of asthma exacerbations | 54,417 |  | 2,120 | 7,185 | 8,604 | 16,046 | 20,462 |  |  |
| Annual number of asthma exacerbations  (n/100,000 persons) | 2,141 |  | 3,537 | 2,249 | 2,319 | 2,154 | 1,956 |  |  |
| **2. Asthma-related hospital utilization** |  |  |  |  |  |  |  |  |  |
| Primary hospital visits | 311,691 (64.5%) |  | 7,203 (62.5%) | 39,091 (64.0%) | 45,081 (64.4%) | 90,524 (64.3%) | 129,792 (65.0%) |  |  |
| Secondary hospital visits | 267,483 (55.4%) |  | 6,404 (55.5%) | 33,679 (55.1%) | 38,804 (55.4%) | 78,108 (55.4%) | 110,488 (55.4%) |  |  |
| Tertiary hospital visits | 232,210 (48.1%) |  | 5,693 (49.4%) | 29,272 (47.9%) | 33,734 (48.2%) | 68,104 (48.3%) | 95,407 (47.8%) |  |  |
| ED visits, patient (n) | 7,272 (1.5%) |  | 259 (2.2%) | 992 (1.6%) | 1,144 (1.6%) | 2,049 (1.5%) | 2,828 (1.4%) |  | <.0001 |
| Admissions, patient (n) | 18,323 (3.8%) |  | 790 (6.8%) | 2,720 (4.5%) | 3,050 (4.4%) | 5,524 (3.9%) | 6,239 (3.1%) |  | <.0001 |
| ICU admission, patient (n) | 25 (0.0%) |  | 6 (0.1%) | 4 (0.0%) | 3 (0.0%) | 5 (0.0%) | 7 (0.0%) |  | <.0001 |
| Annual primary hospital visits, (n/100,000 persons) | 32,586 |  | 29,994 | 31,905 | 32,120 | 32,064 | 33,512 |  | <.0001 |
| Annual Secondary hospital visits, (n/100,000 persons) | 21,519 |  | 21,807 | 21,403 | 21,329 | 21,460 | 21,648 |  |  |
| Annual tertiary hospital visits, (n/100,000 persons) | 15,953 |  | 16,919 | 15,930 | 15,817 | 16,028 | 15,902 |  | <.0001 |
| Annual ED visits, patient, (n/100,000 persons) | 281 |  | 424 | 304 | 302 | 270 | 266 |  | <.0001 |
| Annual hospitalizations, (n/100,000 persons) | 720 |  | 1,344 | 853 | 823 | 741 | 594 |  | <.0001 |
| Annual ICU admission, patient, (n/100,000 persons) | 1 |  | 10 | 1 | 1 | 1 | 1 |  | <.0001 |

ED, emergency department; ICU, intensive care unit; ^a^ Values are reported as *n* (%) unless otherwise indicated; ^b^ Obtained from Korean National Health Insurance Service (NHIS) data

C. ≥12, <19 years of age

|  | **Total**  No. (%), (*n* = 215,359) |  | **Socioeconomic status (SES, 0 to 4)** | | | | |  | ***p*-value** |
| --- | --- | --- | --- | --- | --- | --- | --- | --- | --- |
|  |  |  | **0 (Medical aid)** No. (%),  (*n* = 11,990) | **1 (Lowest)**  No. (%),  (*n* = 33,359) | **2 (Low to Middle)** No. (%),  (*n* = 31,626) | **3 (Middle to High)** No. (%),  (*n* = 47,133) | **4 (Highest)**  No. (%),  (*n* = 91,251) |  |  |
| **1. Asthma exacerbation** |  |  |  |  |  |  |  |  |  |
| Dianosis of asthma exacerbations | 8,449 (3.9%) |  | 701 (5.8%) | 1,410 (4.2%) | 1,344 (4.2%) | 1,849 (3.9%) | 3,145 (3.4%) |  | <.0001 |
| Events numbers of asthma exacerbations | 29,793 |  | 2,744 | 4,763 | 4,468 | 6,432 | 11,386 |  |  |
| Annual number of asthma exacerbations  (n/100,000 persons) | 3,006 |  | 4,924 | 3,110 | 3,061 | 2,960 | 2,717 |  |  |
| **2. Asthma-related hospital utilization** |  |  |  |  |  |  |  |  |  |
| Primary hospital visits | 142,572 (66.2%) |  | 7,233 (60.3%) | 21,927 (65.7%) | 21,257 (67.2%) | 31,449 (66.7%) | 60,706 (66.5%) |  |  |
| Secondary hospital visits | 109,278 (50.7%) |  | 6,057 (50.5%) | 16,863 (50.6%) | 16,237 (51.3%) | 23,955 (50.8%) | 46,166 (50.6%) |  |  |
| Tertiary hospital visits | 87,260 (40.5%) |  | 5,107 (42.6%) | 13,546 (40.6%) | 12,943 (40.9%) | 19,106 (40.5%) | 36,558 (40.1%) |  |  |
| ED visits, patient (n) | 3,683 (1.7%) |  | 365 (3.0%) | 616 (1.8%) | 570 (1.8%) | 827 (1.8%) | 1,305 (1.4%) |  | <.0001 |
| Admissions, patient (n) | 6,596 (3.1%) |  | 730 (6.1%) | 1,184 (3.5%) | 1,059 (3.3%) | 1,496 (3.2%) | 2,127 (2.3%) |  | <.0001 |
| ICU admission, patient (n) | 32 (0.0%) |  | 5 (0.0%) | 8 (0.0%) | 3 (0.0%) | 6 (0.0%) | 10 (0.0%) |  | 0.0524 |
| Annual primary hospital visits, (n/100,000 persons) | 31,787 |  | 29,127 | 31,855 | 30,711 | 31,059 | 33,061 |  | <.0001 |
| Annual Secondary hospital visits, (n/100,000 persons) | 22,737 |  | 21,284 | 22,317 | 22,326 | 23,037 | 22,882 |  |  |
| Annual tertiary hospital visits, (n/100,000 persons) | 17,762 |  | 16,794 | 17,254 | 17,602 | 18,369 | 17,543 |  | <.05 |
| Annual ED visits, patient, (n/100,000 persons) | 332 |  | 529 | 360 | 357 | 324 | 306 |  | <.0001 |
| Annual hospitalizations, (n/100,000 persons) | 1,159 |  | 1,624 | 1,247 | 1,353 | 1,269 | 929 |  | <.0001 |
| Annual ICU admission, patient, (n/100,000 persons) | 1 |  | 8 | 2 | 1 | 1 | 1 |  |  |

ED, emergency department; ICU, intensive care unit; ^a^ Values are reported as *n* (%) unless otherwise indicated; ^b^ Obtained from Korean National Health Insurance Service (NHIS) data

**Supplementary Table 2**. Crude and adjusted hazard ratios (HR) of major treatments, emergency department (ED) visits, and admission to hospital and intensive care unit (ICU) because of childhood asthma, divided by three age groups

**A**. Ventilator support or tracheal intubation, and administration of systemic corticosteroids

|  | | **Ventilator support or tracheal intubation** ^a^ | | | | |  | **Systemic corticosteroid** ^a^ | | | | |
| --- | --- | --- | --- | --- | --- | --- | --- | --- | --- | --- | --- | --- |
|  | | Patient,  No. (%) | cHR  (95% CI) | *p*-value | aHR ^b^  (95% CI) | *p*-value |  | Patient,  No. (%) | cHR  (95% CI) | *p*-value | aHR ^b^  (95% CI) | *p*-value |
| **Total (≥2, <19 years of age) (*N* = 1,537,066)** | | |  |  |  |  |  |  |  |  |  |  |
| SES | 0 (*n* = 35,292) | 5 (0.0) | 4.71 (1.74–12.77) | 0.0023 | 3.73 (1.35–10.35) | 0.0113 |  | 15,266 (43.3) | 1.02 (1.00–1.03) | 0.0439 | 1.04 (1.02–1.06) | <.0001 |
|  | 1 (*n* = 181,756) | 12 (0.0) | 2.24 (1.07–4.69) | 0.0324 | 2.15 (1.03–4.51) | 0.0420 |  | 78,022 (42.9) | 1.02 (1.01–1.03) | <.0001 | 1.02 (1.01–1.03) | <.0001 |
|  | 2 (*n* = 241,103) | 5 (0.0) | 0.70 (0.26–1.90) | 0.4855 | 0.74 (0.27–2.02) | 0.5569 |  | 105,946 (43.9) | 1.05 (1.04–1.06) | <.0001 | 1.04 (1.03–1.05) | <.0001 |
|  | 3 (*n* = 498,685) | 14 (0.0) | 0.96 (0.47–1.95) | 0.9135 | 1.10 (0.54–2.26) | 0.7850 |  | 219,088 (43.9) | 1.05 (1.05–1.06) | <.0001 | 1.04 (1.04–1.05) | <.0001 |
|  | 4 (*n* = 580,230) | 17 (0.0) | Ref |  | Ref |  |  | 244,504 (42.1) | Ref |  | Ref |  |
| **A. ≥2, <6 years of age (***n* = **838,653)** | | |  |  |  |  |  |  |  |  |  |  |
| SES | 0 (*n* = 11,768) | 2 (0.0) | 9.42 (1.83–48.57) | 0.0073 | 10.89 (2.05–57.71) | 0.0050 |  | 5,395 (45.8) | 1.03 (1.00–1.06) | 0.0622 | 1.03 (1.00–1.06) | 0.0583 |
|  | 1 (*n* = 87,328) | 4 (0.0) | 2.63 (0.71–9.81) | 0.1488 | 2.62 (0.70–9.76) | 0.1509 |  | 38,895 (44.5) | 1.02 (1.01–1.03) | 0.0026 | 1.01 (1.00–1.03) | 0.0137 |
|  | 2 (*n* = 139,491) | 3 (0.0) | 1.22 (0.29–5.11) | 0.7844 | 1.11 (0.26–4.70) | 0.8863 |  | 63,508 (45.5) | 1.04 (1.03–1.05) | <.0001 | 1.03 (1.02–1.04) | <.0001 |
|  | 3 (*n* = 310,670) | 4 (0.0) | 0.74 (0.20–2.76) | 0.6557 | 0.71 (0.19–2.65) | 0.6096 |  | 141,460 (45.5) | 1.05 (1.04–1.06) | <.0001 | 1.04 (1.03–1.05) | <.0001 |
|  | 4 (*n* = 289,396) | 5 (0.0) | Ref |  | Ref |  |  | 126,701 (43.8) | Ref |  | Ref |  |
| **B. ≥6,<12 years of age (***n* = **483,054)** | | |  |  |  |  |  |  |  |  |  |  |
| SES | 0 (*n* = 11,534) | 2 (0.0) | 8.63 (1.58–47.14) | 0.0128 | 8.62 (1.54–48.29) | 0.0143 |  | 4,562 (39.6) | 1.00 (0.97–1.03) | 0.8565 | 1.01 (0.98–1.04) | 0.5227 |
|  | 1 (*n* = 61,069) | 2 (0.0) | 1.63 (0.30–8.90) | 0.5726 | 1.64 (0.30–8.94) | 0.5698 |  | 24,392 (39.9) | 1.02 (1.00–1.03) | 0.0428 | 1.02 (1.00–1.03) | 0.0242 |
|  | 2 (*n* = 69,986) | 1 (0.0) | 0.70 (0.08–6.29) | 0.7532 | 0.71 (0.08–6.39) | 0.7575 |  | 28,345 (40.5) | 1.03 (1.02–1.04) | <.0001 | 1.03 (1.01–1.04) | <.0001 |
|  | 3 (*n* = 140,882) | 5 (0.0) | 1.75 (0.47–6.53) | 0.4028 | 1.76 (0.47–6.61) | 0.4011 |  | 56,780 (40.3) | 1.03 (1.01–1.04) | <.0001 | 1.03 (1.01–1.04) | <.0001 |
|  | 4 (*n* = 199,583) | 4 (0.0) | Ref |  | Ref |  |  | 78,512 (39.3) | Ref |  | Ref |  |
| **C. ≥12, <19 years of age (***n* = **215,359)** | | |  |  |  |  |  |  |  |  |  |  |
| SES | 0 (*n* = 11,990) | 1 (0.0) | 0.93 (0.12–7.44) | 0.9456 | 0.88 (0.11–7.10) | 0.9013 |  | 5,309 (44.3) | 1.01 (0.99–1.04) | 0.3307 | 1.01 (0.98–1.04) | 0.5638 |
|  | 1 (*n* = 33,359) | 6 (0.0) | 2.04 (0.71–5.88) | 0.1865 | 2.06 (0.71–5.93) | 0.1816 |  | 14,735 (44.2) | 1.03 (1.01–1.05) | 0.0036 | 1.03 (1.01–1.05) | 0.0061 |
|  | 2 (*n* = 31,626) | 1 (0.0) | 0.36 (0.05–2.86) | 0.3324 | 0.37 (0.05–2.95) | 0.3469 |  | 14,093 (44.6) | 1.04 (1.02–1.06) | <.0001 | 1.04 (1.02–1.06) | <.0001 |
|  | 3 (*n* = 47,133) | 5 (0.0) | 1.20 (0.39–3.68) | 0.7464 | 1.26 (0.41–3.89) | 0.6838 |  | 20,848 (44.2) | 1.03 (1.02–1.05) | 0.0002 | 1.04 (1.02–1.05) | <.0001 |
|  | 4 (*n* = 91,251) | 8 (0.0) | Ref |  | Ref |  |  | 39,291 (43.1) | Ref |  | Ref |  |

cHR, crude hazard ratio; aHR, adjusted hazard ratio; Ref, reference; ^a^ Obtained from Korean National Health Insurance Service (NHIS) data, ^b^ adjusted by age, sex, type of insurance, allergy history, respiratory disease (bronchiolitis, croup, pneumonia, and *Mycoplasma pneumoniae* infections)

**B**. Emergency department visits

|  | | **Emergency department (ED) visits** ^a^ | | | | |
| --- | --- | --- | --- | --- | --- | --- |
|  | | Patient,  No. (%) | cHR  (95% CI) | *p*-value | aHR ^b^  (95% CI) | *p*-value |
| **Total (≥2, <19 years of age) (*N* = 1,537,066)** | | |  |  |  |  |
| SES | 0 (*n* = 35,292) | 932 (2.6) | 1.75 (1.63–1.87) | <.0001 | 1.88 (1.75–2.01) | <.0001 |
|  | 1 (*n* = 181,756) | 3,203 (1.8) | 1.18 (1.13–1.23) | <.0001 | 1.19 (1.14–1.24) | <.0001 |
|  | 2 (*n* = 241,103) | 4,238 (1.8) | 1.17 (1.13–1.22) | <.0001 | 1.15 (1.11–1.19) | <.0001 |
|  | 3 (*n* = 498,685) | 7,817 (1.6) | 1.06 (1.03–1.09) | 0.0004 | 1.04 (1.01–1.07) | 0.0099 |
|  | 4 (*n* = 580,230) | 8,635 (1.5) | Ref |  | Ref |  |
| **A. ≥2, <6 years of age (*n* = 838,653)** | | |  |  |  |  |
| SES | 0 (*n* = 11,768) | 308 (2.6) | 1.63 (1.45–1.83) | <.0001 | 1.64 (1.46–1.84) | <.0001 |
|  | 1 (*n* = 87,328) | 1,595 (1.8) | 1.17 (1.10–1.24) | <.0001 | 1.16 (1.09–1.23) | <.0001 |
|  | 2 (*n* = 139,491) | 2,524 (1.8) | 1.15 (1.09–1.20) | <.0001 | 1.10 (1.05–1.15) | 0.0002 |
|  | 3 (*n* = 310,670) | 4,941 (1.6) | 1.02 (0.98–1.06) | 0.3847 | 1.00 (0.96–1.04) | 0.9175 |
|  | 4 (*n* = 289,396) | 4,502 (1.6) | Ref |  | Ref |  |
| **B. ≥6,<12 years of age (*n* = 483,054)** | | |  |  |  |  |
| SES | 0 (*n* = 11,534) | 259 (2.2) | 1.59 (1.40–1.81) | <.0001 | 1.69 (1.49–1.92) | <.0001 |
|  | 1 (*n* = 61,069) | 992 (1.6) | 1.15 (1.07–1.23) | 0.0002 | 1.15 (1.07–1.24) | 0.0002 |
|  | 2 (*n* = 69,986) | 1,144 (1.6) | 1.15 (1.07–1.23) | <.0001 | 1.13 (1.05–1.21) | 0.0008 |
|  | 3 (*n* = 140,882) | 2,049 (1.5) | 1.02 (0.97–1.08) | 0.4750 | 1.01 (0.95–1.07) | 0.7497 |
|  | 4 (*n* = 199,583) | 2,828 (1.4) | Ref |  | Ref |  |
| **C. ≥12, <19 years of age (*n* = 215,359)** | | |  |  |  |  |
| SES | 0 (*n* = 11,990) | 365 (3.0) | 2.10 (1.87–2.36) | <.0001 | 2.21 (1.96–2.48) | <.0001 |
|  | 1 (*n* = 33,359) | 616 (1.8) | 1.29 (1.17–1.42) | <.0001 | 1.29 (1.17–1.42) | <.0001 |
|  | 2 (*n* = 31,626) | 570 (1.8) | 1.25 (1.13–1.38) | <.0001 | 1.22 (1.11–1.35) | <.0001 |
|  | 3 (*n* = 47,133) | 827 (1.8) | 1.22 (1.12–1.33) | <.0001 | 1.19 (1.09–1.30) | <.0001 |
|  | 4 (*n* = 91,251) | 1,305 (1.4) | Ref |  | Ref |  |

cHR, crude hazard ratio; aHR, adjusted hazard ratio; ED, emergency department; Ref, reference, ^a^ Obtained from the Korean National Health Insurance Service (NHIS) data, ^b^ adjusted by age, sex, type of insurance, allergy history, respiratory disease (bronchiolitis, croup, pneumonia, and *Mycoplasma pneumoniae* infections)

**C**. Admission to hospital and ICU

|  | | **Admission to hospital** ^a^ | | | | |  | **Admission to ICU** ^a^ | | | | |
| --- | --- | --- | --- | --- | --- | --- | --- | --- | --- | --- | --- | --- |
|  | | Patient,  No. (%) | cHR  (95% CI) | *p*-value | aHR ^b^  (95% CI) | *p*-value |  | Patient,  No. (%) | cHR  (95% CI) | *p*-value | aHR ^b^  (95% CI) | *p*-value |
| **Total (≥2, <19 years of age) (*n* = 1,537,066)** | | |  |  |  |  |  |  |  |  |  |  |
| SES | 0 (*n* = 35,292) | 2,734 (7.7) | 1.76 (1.69–1.83) | <.0001 | 2.20 (2.11–2.28) | <.0001 |  | 14 (0.0) | 7.02 (3.75–13.16) | <.0001 | 7.12 (3.72–13.62) | <.0001 |
|  | 1 (*n* = 181,756) | 10,737 (5.9) | 1.34 (1.31–1.38) | <.0001 | 1.37 (1.34–1.40) | <.0001 |  | 19 (0.0) | 1.89 (1.07–3.33) | 0.0281 | 1.85 (1.05–3.27) | 0.0333 |
|  | 2 (*n* = 241,103) | 15,462 (6.4) | 1.46 (1.43–1.49) | <.0001 | 1.34 (1.31–1.36) | <.0001 |  | 17 (0.0) | 1.27 (0.71–2.29) | 0.4274 | 1.23 (0.68–2.22) | 0.4985 |
|  | 3 (*n* = 498,685) | 29,597 (5.9) | 1.36 (1.34–1.38) | <.0001 | 1.21 (1.19–1.24) | <.0001 |  | 22 (0.0) | 0.80 (0.47–1.38) | 0.4272 | 0.83 (0.48–1.43) | 0.4899 |
|  | 4 (*n* = 580,230) | 25,634 (4.4) | Ref |  | Ref |  |  | 32 (0.0) | Ref |  | Ref |  |
| **A. ≥2, <6 years of age (*N* = 838,653)** | | |  |  |  |  |  |  |  |  |  |  |
| SES | 0 (*n* = 11,768) | 1,214 (10.3) | 1.72 (1.63–1.83) | <.0001 | 1.77 (1.67–1.88) | <.0001 |  | 3 (0.0) | 4.73 (1.37–16.33) | 0.0140 | 5.36 (1.53–18.77) | 0.0087 |
|  | 1 (*n* = 87,328) | 6,833 (7.8) | 1.32 (1.28–1.36) | <.0001 | 1.31 (1.27–1.35) | <.0001 |  | 7 (0.0) | 1.54 (0.63–3.77) | 0.3478 | 1.53 (0.62–3.76) | 0.3518 |
|  | 2 (*n* = 139,491) | 11,353 (8.1) | 1.36 (1.33–1.40) | <.0001 | 1.30 (1.27–1.33) | <.0001 |  | 11 (0.0) | 1.50 (0.69–3.26) | 0.3108 | 1.35 (0.62–2.96) | 0.4557 |
|  | 3 (*n* = 310,670) | 22,577 (7.3) | 1.22 (1.20–1.25) | <.0001 | 1.18 (1.16–1.20) | <.0001 |  | 11 (0.0) | 0.68 (0.31–1.48) | 0.3311 | 0.66 (0.30–1.43) | 0.2901 |
|  | 4 (*n* = 289,396) | 17,268 (6.0) | Ref |  | Ref |  |  | 15 (0.0) | Ref |  | Ref |  |
| **B. ≥6,<12 years of age (*n* = 483,054)** | | |  |  |  |  |  |  |  |  |  |  |
| SES | 0 (*n* = 11,534) | 790 (6.8) | 2.24 (2.08–2.41) | <.0001 | 2.43 (2.25–2.61) | <.0001 |  | 6 (0.1) | 14.85 (4.99–44.19) | <.0001 | 17.23 (5.61–52.90) | <.0001 |
|  | 1 (*n* = 61,069) | 2,720 (4.5) | 1.44 (1.37–1.50) | <.0001 | 1.43 (1.37–1.50) | <.0001 |  | 4 (0.0) | 1.87 (0.55–6.38) | 0.3188 | 1.87 (0.55–6.38) | 0.3198 |
|  | 2 (*n* = 69,986) | 3,050 (4.4) | 1.39 (1.33–1.46) | <.0001 | 1.34 (1.29–1.40) | <.0001 |  | 3 (0.0) | 1.21 (0.31–4.69) | 0.7800 | 1.14 (0.29–4.45) | 0.8528 |
|  | 3 (*n* = 140,882) | 5,524 (3.9) | 1.25 (1.21–1.30) | <.0001 | 1.22 (1.17–1.26) | <.0001 |  | 5 (0.0) | 1.01 (0.32–3.17) | 0.9922 | 0.95 (0.30–3.00) | 0.9273 |
|  | 4 (*n* = 199,583) | 6,239 (3.1) | Ref |  | Ref |  |  | 7 (0.0) | Ref |  | Ref |  |
| **C. ≥12, <19 years of age (*n* = 215,359)** | | |  |  |  |  |  |  |  |  |  |  |
| SES | 0 (*n* = 11,990) | 730 (6.1) | 2.63 (2.41–2.86) | <.0001 | 2.87 (2.63–3.12) | <.0001 |  | 5 (0.0) | 3.73 (1.28–10.92) | 0.0162 | 4.12 (1.37–12.37) | 0.0117 |
|  | 1 (*n* = 33,359) | 1.184 (3.5) | 1.53 (1.42–1.64) | <.0001 | 1.53 (1.42–1.64) | <.0001 |  | 8 (0.0) | 2.18 (0.86–5.52) | 0.1005 | 2.19 (0.87–5.56) | 0.0978 |
|  | 2 (*n* = 31,626) | 1.059 (3.3) | 1.44 (1.33–1.55) | <.0001 | 1.39 (1.29–1.50) | <.0001 |  | 3 (0.0) | 0.86 (0.24–3.12) | 0.8165 | 0.84 (0.23–3.06) | 0.7902 |
|  | 3 (*n* = 47,133) | 1.496 (3.2) | 1.36 (1.28–1.46) | <.0001 | 1.30 (1.22–1.39) | <.0001 |  | 6 (0.0) | 1.16 (0.42–3.18) | 0.7800 | 1.11 (0.40–3.08) | 0.8389 |
|  | 4 (*n* = 91,251) | 2.127 (2.3) | Ref |  | Ref |  |  | 10 (0.0) | Ref |  | Ref |  |

cHR, crude hazard ratio; aHR, adjusted hazard ratio; ICU, intensive care unit; Ref, reference; ^a^ Obtained from Korean National Health Insurance Service (NHIS) data, ^b^ adjusted by age, sex, type of insurance, allergy history, respiratory disease (bronchiolitis, croup, pneumonia, and *Mycoplasma pneumoniae* infections)

**Appendix**

**Appendix 1.** International Classification of Diseases (ICD)-10 diagnostic codes of diagnosis and conditions of study subjects.

| Diagnosis and conditions | ICD-10 code |
| --- | --- |
| Prematurity | P07 |
| Congenital malformations or deformations | Q00-Q99 |
| Medical conditions with onset in the perinatal period | P00-P96 |
| Chronic respiratory disease | J43, J44, J47 |
| Factors influencing health status and contact with health services | P10-P159 |
